# Simvastatin may induce insulin resistance through a novel fatty acid mediated cholesterol independent mechanism

**DOI:** 10.1038/srep13823

**Published:** 2015-09-08

**Authors:** Vasundhara Kain, Bandish Kapadia, Parimal Misra, Uday Saxena

**Affiliations:** 1 Department of Biology, Dr. Reddy’s Institute of Life Sciences, University of Hyderabad Campus, Hyderabad, Telangana, India; 2 Kareus Therapeutics, SA, Switzerland

## Abstract

Statins are a class of oral drugs that are widely used for treatment of hypercholesterolemia. Recent clinical data suggest that chronic use of these drugs increases the frequency of new onset diabetes. Studies to define the risks of statin-induced diabetes and its underlying mechanisms are clearly necessary. We explored the possible mechanism of statin induced insulin resistance using a well-established cell based model and simvastatin as a prototype statin. Our data show that simvastatin induces insulin resistance in a cholesterol biosynthesis inhibition independent fashion but does so by a fatty acid mediated effect on insulin signaling pathway. These data may help design strategies for prevention of statin induced insulin resistance and diabetes in patients with hypercholesterolemia.

Statins are a class of drugs that are used for lowering of plasma cholesterol. There are several marketed statins and they work by inhibiting 3-hydroxy-3-methylglutaryl-coenzyme A (HMG-CoA) reductase, an intracellular enzyme which plays a central role in production of cholesterol^1^,^2^,^3^,^4^,^5^. Most statins are similar in structure to the enzyme’s substrate, HMG-CoA and act as competitive inhibitors. Inhibition of this enzyme by statins translates into lowering of circulating cholesterol enriched low density lipoproteins (LDL) levels. High LDL levels are causally associated with increased cardiovascular disease (CVD)^6^,^7^,^8^,^9^.

Statins are used as front line therapy for lowering plasma cholesterol and prevention of CVD. Several clinical studies have shown that statins are very effective in reducing death and disability due to CVD^10^,^11^,^12^,^13^,^14^,^15^,^16^,^17^. Recent clinical data however now show that chronic statin therapy is associated with increased risk and occurrence of insulin resistance and type 2 diabetes^18^,^19^,^20^,^21^. For example, analysis of data from six large clinical studies of patients using statins has led to revelation that there is a 13% increased risk of new onset diabetes in patients on statin therapy^22^. Similarly another study using data from over ninety thousand patients on statin therapy showed a 12% increase in propensity of new onset diabetes^23^,^24^. These data linking statins with type 2 diabetes are especially concerning since this class of drugs are used by millions of patients in the USA as well as in the rest of the world^25^. Therefore since 2012, the US Food and Drug Administration requires statin drug package insert to include a warning about the risk of type 2 diabetes with its use^26^.

Given that statins are chronically used by millions of patients worldwide, it is important to understand the mechanism of increased incidence of diabetes^25^. Some studies have probed this mechanism. Cell based studies have suggested that statins may interfere with pancreatic β-cell insulin secretion by either decreasing Ca^2+^-dependent insulin secretion or by interfering with isoprenylation of guanosine triphosphate(GTP)–binding proteins^27^. Other studies suggest that statin inhibition of isoprenoid biosynthesis may lead to lower expression of insulin signaling proteins in adipocytes and to reduced glucose transporter expression or translocation^28^.

Data reported in another study attempted to examine the mechanism in patients. This study connects the effect of simvastatin treatment with levels of Uncoupling protein (UCP3)^29^. The results inversely correlated the levels of UCP3 in simvastatin treated patients with degree of insulin resistance. It was shown that statin treatment lowers UCP3 levels. Using oral glucose tolerance test it was further shown that insulin resistance correlated with the decrease in UCP-3 caused by simvastatin. Since UCP3 inhibits the accumulation of intracellular free fatty acids (FFAs), it was proposed that this accumulation of FFAs may be the reason for statin induced insulin resistance^29^.

The *in vitro* studies mentioned above used either adipocytes or pancreatic β-cells as model systems to understand the mechanism of statin induced diabetes. Since a major hallmark of type 2 diabetes is insulin resistance, we decided to use muscle cell based system to understand the mechanism. Muscle accounts for major portion of whole body glucose uptake and any impairment in glucose uptake by this tissue may result in onset of diabetes. Our results show that a novel fatty acid dependent, cholesterol synthesis independent mechanism of statins may lead to insulin resistance and increased incidence of diabetes.

## Results

### Simvastatin blocks insulin mediated glucose uptake

**Figure 1:**
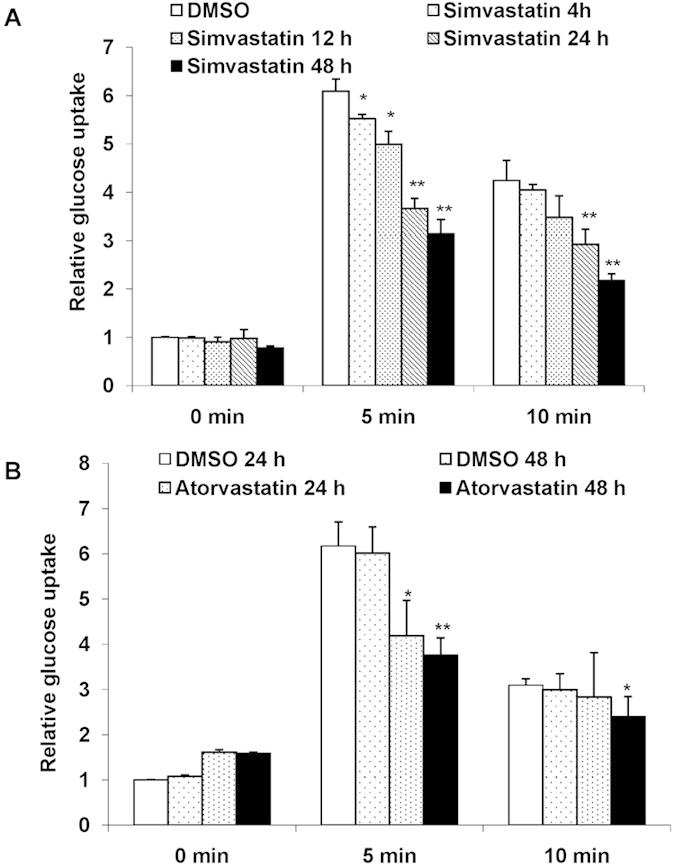
Simvastatin and Atorvastatin block insulin mediated glucose uptake: Mean basal and insulin stimulated (5 min and 10 min) uptake of 2-NBDG by L6 myotubes treated with Simvastatin (1 μM) (**a**) and Atorvastatin (**b**) for different time points as indicated. DMSO (0.1%) treated cells served as internal control. Values are shown as mean±SD after normalizing with the corresponding protein content and expressed relative to basal of control cells which was set to 1; *p < 0.05 ,**p < 0.01, versus corresponding control cells (two way ANOVA).

To explore if simvastatin could induce insulin resistance like phenotype in an *in vitro* model, we explored its effects on the uptake of glucose using a L_6_ myotubes model (Supplemental Fig. 1). The effect of simvastatin on glucose uptake was observed in myotubes with and without treatment with insulin using 2-NBDG as a marker for glucose uptake. Addition of simvastatin to control cells (no treatment with insulin) had minimal effect on glucose uptake (Supplemental Fig. 1). However addition of simvastatin followed by insulin stimulation, markedly inhibited glucose uptake (~5% after 4 h, ~8% after 12 h, ~40% after 24 h and ~60% after 48 h of treatment compared to DMSO treated control cells post 5minutes of stimulation with insulin) (Fig. 1a). We also examined the effects of atorvastatin, another statin that is clinically used but its chemical structure is distinct from that of simvastatin. As shown in Fig. 1b, atorvastatin treatment also reduced insulin stimulated glucose uptake. These data show that two distinct statins, simvastatin and atorvastatin can inhibit insulin-mediated uptake of glucose. Prolonged simvastatin treatment (48 h) showed marginal decrease in glucose uptake of control cells, while atorvastatin displayed modest increase in glucose uptake of control cells (statistically non significant). Collectively these data show that these two statins impair insulin-mediated but not non-insulin mediated glucose uptake.

### Mevalonate is unable to rescue simvastatin induced inhibition of glucose uptake

**Figure 2:**
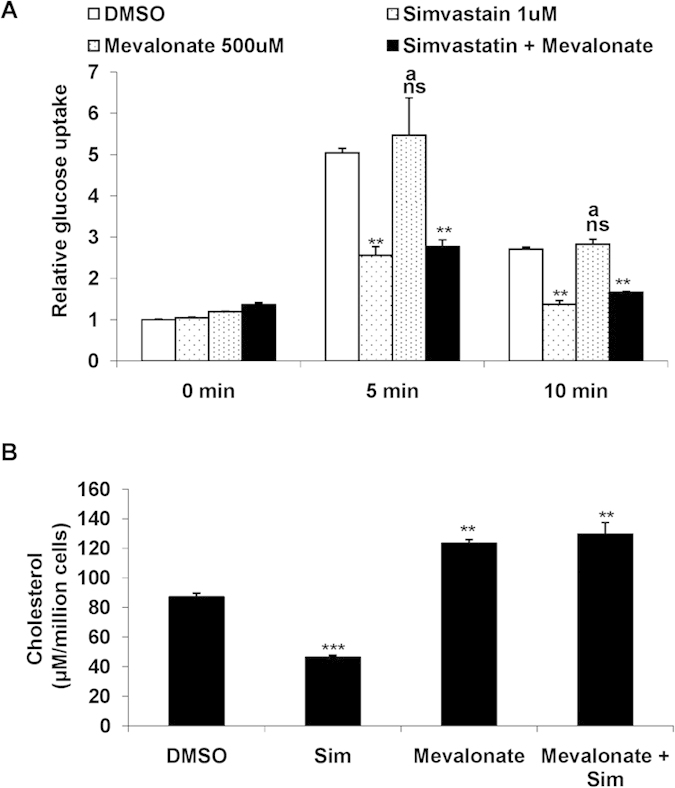
(**a**) Mevalonate is unable to rescue Simvastatin induced inhibition of glucose uptake: Mean basal and insulin stimulated (5 min and 10 min) uptake of 2-NBDG by L6 myotubes treated with Simvastatin (1 μM) and/or Mevalonate (500 μM) for 48 h. DMSO (0.1%) treated cells served as internal control. Values are shown as mean±SD after normalizing with the corresponding protein content and expressed relative to basal of control cells which was set to 1; **p < 0.01, versus corresponding control cells , ^ns^non significant, ^a^*p *< 0.01 versus corresponding Simvastatin treated cells (two way ANOVA). (**b**) Total Cholesterol levels were depleted upon Simvastatin treatment: L6 myotubes were cultured for 48 h in presence of Simvastatin (1 μM) or Mevalonate (500 μM). Post treatment lipid was extracted and total cholesterol was estimated. Values were further normalized with the total cell count and represented as μM per million cells. **p < 0.01, ***p < 0.005 versus corresponding control (DMSO: 0.1%) cells, ^b^*p *< 0.001 versus Simvastatin (1 μM) treated cells (two way ANOVA).

We next explored if the inhibition of insulin mediated glucose uptake by simvastatin was related to the drug’s ability to inhibit intracellular cholesterol biosynthesis. It is known that the addition of excess mevalonate, a precursor for cholesterol biosynthesis, rescues the cells from statin’s cholesterol synthesis inhibitory effects. Therefore, we treated cells with simvastatin together with mevalonate and then examined the effect of simvastatin on insulin mediated glucose uptake for 5 min and 10 min. Incubation with mevalonate failed to rescue the cells from the inhibitory effect of simvastatin on insulin mediated glucose uptake (~50% reduction in glucose uptake with simvastatin alone and co-treatment with simvastatin and mevalonate) (Fig. 2a). To make sure that mevalonate treatment did increase cholesterol synthesis we also examined cholesterol content in the cells. As shown in Fig. 2b, simvastatin treatment alone as expected reduced cellular cholesterol content by about 50% relative to untreated cells, whereas when mevalonate was added together with simvastatin, the cholesterol content increased to slightly higher than that observed in control cells. Addition of mevalonate to control cells also increased cholesterol content. This suggests that the inhibitory effect of simvastatin on insulin stimulated glucose may be unrelated to its ability to block cellular cholesterol biosynthesis pathway.

### Simvastatin does not impair Peroxisome Proliferator Activated Receptor (PPAR) gamma mediated glucose uptake

We then explored if simvastatin may have caused general defect(s) in signaling pathways related to glucose uptake by depleting the cellular membranes of cholesterol. To this end, we examined if the drug had any impact on PPAR gamma mediated glucose uptake, a critical pathway in glucose regulation in cells and tissues.

**Figure 3:**
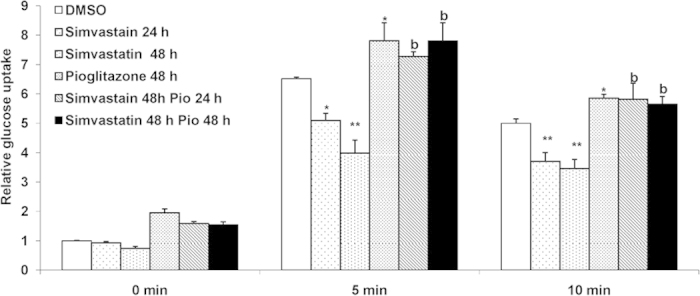
Simvastatin does not impair PPAR-γ mediated glucose uptake: Mean basal and insulin stimulated (5 min and 10 min) uptake of 2-NBDG by L6 myotubes treated with Simvastatin (1 μM) and/or Pioglitazone (10 μM) for different time points as indicated. DMSO (0.1%) treated cells served as internal control. Values are shown as mean±SD after normalizing with the corresponding protein content and expressed relative to basal of control cells which was set to 1; *p < 0.05, **p < 0.01, versus corresponding control cells , ^b^*p *< 0.005 versus corresponding Simvastatin treated cells (two way ANOVA).

Cells were treated either with simvastatin or pioglitazone alone or with a combination of the two drugs. Pioglitazone is a known PPAR gamma activator. As shown in Fig. 3, simvastatin alone had little effect on non-insulin stimulated glucose uptake. But combination of simvastatin with pioglitazone or pioglitazone alone showed substantial (~200% compared to DMSO treated control cells) increase in glucose uptake.

Similarly when the cells were stimulated with insulin, simvastatin alone decreased (~50% decrease compared to DMSO treated insulin stimulated cells) glucose uptake. However in cells that were treated with both simvastatin and pioglitazone, the inhibitory effect of the simvastatin was relieved by pioglitazone and the increase in glucose uptake was similar to that obtained by treatment by pioglitazone alone (Fig. 3). These data show that simvastatin treatment does not impair the PPAR gamma mediated glucose uptake pathway in muscle cells.

We also explored if simvastatin treatment had any impact on the expression of glucose transporter-4 (Glut-4) protein. Glut-4 is a transport protein which in response to insulin treatment transports glucose by translocation. Our results showed (data not shown) that simvastatin treatment had no effect on Glut-4 protein expression suggesting that reduced expression of Glut-4 protein is not the mechanism of simvastatin’s inhibitory effect.

### Simvastatin affects insulin signaling pathway

**Figure 4:**
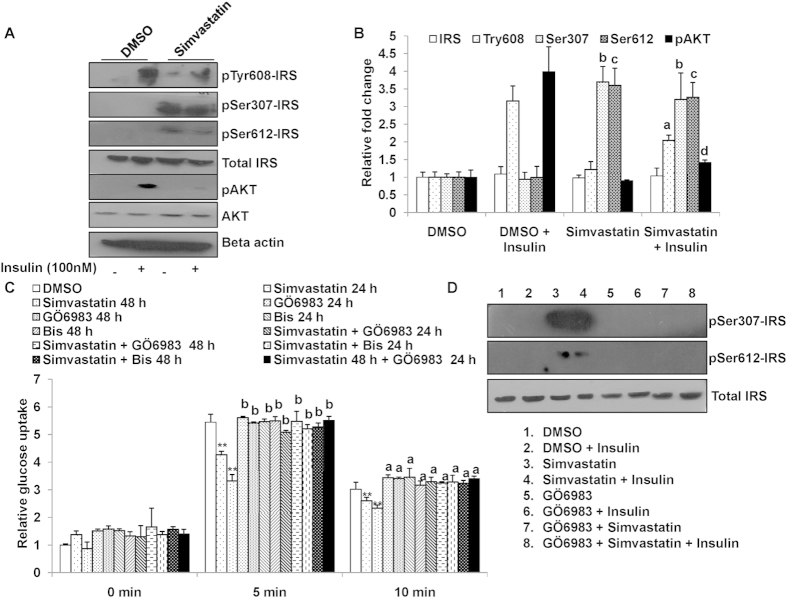
(**a**) Simvastatin treatment inhibits insulin signaling cascade: L6 myotubes were cultured in Simvastatin for 48 h, followed by stimulation with insulin for 20 min. Cells were lysed and probed for phosphorylation of IRS-1 (Ser^307^and Ser^612^, Tyr^608^) and pAKT. Total IRS-1, AKT and Beta-actin served as loading control. The cropped blots were run under the same experimental conditions. The full-length blots are included in Supplemental Figure 4. (**b**) Densitometric quantification results for western blots from Fig. 4A are shown. Values are shown as mean ± SD after normalizing with the corresponding protein content and expressed relative to basal (DMSO) of control cells which was set to 1; ^a,d^*p *< 0.05 versus DMSO + insulin treated cells, ^b,c^*p *< 0.005 versus corresponding DMSO treated cells (two way ANOVA). (**c**) Inhibition of PKC rescues Simvastatin inhibited insulin mediated glucose uptake: Mean basal and insulin stimulated (5 min and 10 min) uptake of 2-NBDG by L6 myotubes treated with Simvastatin (1 μM) and/or PKC inhibitors (Bisindolylmaleimide I, Bis (1 μM) and GÖ6983 (1 μM) for different time points as indicated. DMSO (0.1%) treated cells served as internal control. Values are shown as mean ± SD after normalizing with the corresponding protein content and expressed relative to basal of control cells which was set to 1; **p < 0.01, versus corres*p*onding control cells, ^b^*p *< 0.005 versus corresponding Simvastatin treated cells (two way ANOVA). (**d**) Pharmaocological Inhibition of PKC pathway diminished simvastatin induced IRS serine phosphorylation. L6 myotubes were cultured in Simvastatin (1 μM) and/or GÖ6983 (1 μM) for 48 h, followed by stimulation with insulin for 20 min. Cells were lysed and probed for phosphorylation of IRS-1 (Ser^307^and Ser^612^) and total IRS-1. The cropped blots were run under the same experimental conditions. The full-length blots are included in Supplemental Figure 4.

Since initial studies showed that simvastatin only affects insulin stimulated glucose uptake but not un-stimulated uptake (Fig. 1) we explored whether the drug may have a direct effect on insulin signaling. Insulin receptor substrate- 1 (IRS-1) plays a key role in transmitting signals from the insulin receptors to intracellular PI3K/Akt pathways. Binding of insulin to its cell-surface receptor triggers the intracellular phosphorylation of Tyr^608^ in IRS-1 and glucose uptake by Glut-4 transporter translocation. Conversely, inhibition of its phosphorylation of Tyr^608^ in IRS-1 impedes glucose uptake. To explore simvastatin’s effects on IRS-1 phosphorylation, cells were treated with and without simvastatin and were incubated with insulin for 20 min followed by measurement of IRS-1 phosphorylation at Tyr^608^ by western blotting. As shown in Fig. 4a, under insulin stimuli, simvastatin treatment significantly inhibited IRS-1 phosphorylation at Tyr^608^ (~50%) and pAKT (~70%) compared to DMSO treated control cells (Fig. 4b). These data suggest that simvastatin may inhibit insulin mediated glucose uptake by blocking a critical steps in insulin signaling cascade.

We then further explored if simvastatin may be inhibiting insulin signaling via a protein kinase C (PKC) mediated step. It is well established that events such as accumulation of cellular free fatty acids (FFA) increase intracellular level of diacyl glycerol (DAG), which allosterically activates PKC^30^. This activated PKC phosphorylates IRS at Ser^307^ and Ser^612^ which in turn hampers Tyr^608^ phosphorylation and inhibits insulin stimulated glucose uptake. So, we next measured the phosphorylation status of Ser^307^ and Ser^612^ in IRS-1 post simvastatin treatment. Treatment with simvastatin followed by insulin stimulus robustly increased Ser^307^ and Ser^612^ phosphorylation of IRS-1 (Fig. 4a,b).

Furthermore, we incubated the cells in presence or absence of a pan PKC inhibitor (bisindolylmaleimide I^31^) together with simvastatin for 24 h and 48 h followed by insulin stimulation for 5 and 10 min. Inhibition of PKC reversed the effect of simvastatin and resulted in a ~100% and~250% increase (for 24 h and 48 h in co-treated cells) compared to simvastatin treatment alone (Fig. 4b). These data demonstrate that inhibiting PKC completely abolishes simvastatin’s effects on insulin signaling.

Since bisindolylmaleimide I^31^ is a pan PKC inhibitor, we also examined the effects of a selective PKC inhibitor GÖ6983. As shown in Fig. 4b, this selective inhibitor of PKC also reversed the effects of simvastatin. Concurrently the serine phosphorylation status of IRS^307^ and IRS^612^ were markedly decreased with treatment of GÖ6983 in simvastatin treated L6 myotubes (Fig. 4d, Supplemental Figure 3). These results indicate that simvastatin 1) directly effects insulin signaling pathway 2) simvastatin effect may involve a PKC mediated step.

### Simvastatin induces inhibition of glucose uptake by free fatty acid mediated pathway

Our results from above suggested that simvastatin inhibits insulin signaling via a PKC mediated pathway and FFA accumulation is a known modulator of PKC pathway. We therefore further explored if simvastatin may increase FFA levels in muscle cells. One of the by-products from statin inhibition of HMG CoA reductase is accumulation of acyl-CoA. Two units of acetyl- CoA are converted by thiolase to acetoacetly CoA which is then converted by HMG CoA^32^. HMG CoA is acted upon by HMG CoA reductase (the target enzyme of statins) to produce mevalonic acid^33^. When HMG CoA reductase is inhibited it could lead to acetyl-CoA accumulation in the cells. This accumulation could lead to fatty acid synthesis since acetyl-CoA is the precursor of fatty acids. Thus, we postulated that simvastatin treatment may lead to accumulation of FFAs in muscle leading to insulin resistance.

**Figure 5:**
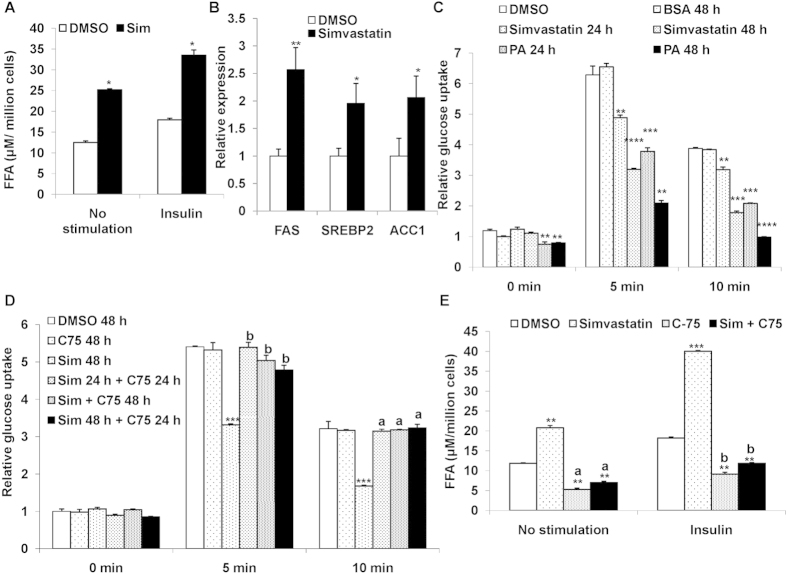
(**a**) Treatment of Simvastatin builds up FFAs in muscle cells: L6 myotubes were cultured for 48 h in presence of Simvastatin (1 μM) followed by treatment with insulin (100 nM) for 20 min. Post treatment lipid was extracted and FFAs was estimated. Values were further normalized with the total cell count and represented as μM per million cells. *p < 0.05, versus corresponding control cells (Student’s *t*-test). (**b**) Simvastatin treatment enhances FAS, SREBP2 and ACC1 gene expression: mRNA expression of rattus FAS, SREBP2 and ACC1 in L6 myotubes treated Simvastatin (1 μM) for 48 h. *p < 0.05, **p < 0.01, versus corres*p*onding control cells (Student’s *t*-test). (**c**) Palmitic acid mimics Simvastatin inhibited insulin mediated glucose uptake: Mean basal and insulin stimulated (5 min and 10 min) uptake of 2-NBDG by L6 myotubes treated with Simvastatin (1 μM) or Palmitic acid (PA, 150 μM) for different time points as indicated. DMSO (0.1%) and BSA treated cells served as internal control. Values are shown as mean ± SD after normalizing with the corresponding protein content and expressed relative to basal of control cells (DMSO) which was set to 1; **p < 0.01, ***p < 0.005, ****p < 0.001 versus corres*p*onding control cells (two way ANOVA). (**d**) Inhibition of FAS by C-75 rescues Simvastatin inhibited insulin mediated glucose uptake: Mean basal and insulin stimulated (5 min and 10 min) uptake of 2-NBDG by L6 myotubes treated with Simvastatin (1 μM) and/or FAS inhibitor (C-75, 40 μg/mL) for different time points as indicated. DMSO (0.1%) served as internal control. Values are shown as mean ± SD after normalizing with the corresponding protein content and expressed relative to basal of control cells (DMSO) which was set to 1; ***p < 0.005, versus corresponding control cells, ^a^*p *< 0.01, ^b^*p *< 0.005 versus Simvastatin (1 μM) treated cells (two way ANOVA). (**e**) Inhibition of FAS by C-75 reduces FFAs in Simvastatin treated cells: L6 myotubes were cultured for 48 h in presence of Simvastatin (1 μM) and/or FAS inhibitor (C-75, 40 μg/mL) followed by treatment with insulin (100 nM) for 20 min. Post treatment lipid was extracted and FFAs was estimated. Values were further normalized with the total cell count and represented as μM per million cells. **p < 0.01,***p < 0.001 versus corresponding control cells, ^a^*p *< 0.01, ^b^*p *< 0.005 versus Simvastatin (1 μM) treated cells (two way ANOVA).

To assess our postulation, we examined if simvastatin treatment could lead to FFAs accumulation in the cells. Cells were treated with simvastatin and intracellular FFAs concentrations were estimated. As shown in Fig. 5a, FFAs levels were markedly increased by about 80% with simvastatin treatment in both control (increase from12 μM to 25 μM FFAs) and insulin treated cells (increase from 18 μM to 34 μM FFAs).

Statin treatment is known to induce the expression of SREBP2 and related genes such as fatty acid synthase (FAS) and acetyl coA carboxylase 1 (ACC1)^34^. We also examined the effect of this statin on the expression of these genes. As shown in Fig. 5b, simvastatin treatment did result in 2–2.5-fold induction of all three genes. These data suggest that indication of these genes especially FAS may contribute increased FFAs synthesis and resulting in impact on insulin treated glucose uptake.

We next questioned if addition of exogenous fatty acids could mimic the inhibition of insulin-mediated glucose by simvastatin. We cultured cells in presence of simvastatin alone or palmitic acid alone (150 μM) for 48 h and measured glucose uptake. Indeed, incubation of cells with palmitic acid resulted in significant inhibition (PA: ~50% and 70% reduction for 24 h and 48 h, Fig. 5c) of insulin-mediated uptake, similar to inhibition observed by simvastatin treatment (Fig. 5c).

To further confirm potential role for FFAs in simvastatin inhibited insulin dependent glucose uptake, we cultured cells with simvastatin or compound C-75, a known fatty acid synthase (FAS) inhibitor^35^ or both for 24 h. As expected simvastatin decreased insulin stimulated uptake by 50%, but addition of compound C-75 together with simvastatin increased glucose uptake by ~50% compared to simvastatin alone, similar to DMSO treated cells (Fig. 5d). Thus inhibiting FAS completely negated the statins inhibitory effect on insulin mediated glucose uptake.

We then measured the FFAs concentration in the muscle cells treated with simvastatin or C-75 alone or in combination. Upon inhibition of FAS, there was ~50% reduction in FFAs levels in the control cells (Fig. 5e). On the other hand simvastatin treatment in presence of C-75 did not lead to accumulation of FFAs with or without insulin stimulation (Fig. 5e). This suggests that that simvastatin treated increase in FFAs is responsible for blocking glucose uptake and the FFAs accumulation may be mediated through FAS pathway.

Collectively these studies support the suggestion that simvastatin induced buildup of free fatty acids in the cells could be responsible for its inhibitory effect.

## Discussion

Since the mechanism of statin induced diabetes is not completely understood, we examined potential causes using a muscle cell based model system. Our data shown here demonstrate the following observations: a) Simvastatin inhibits insulin stimulated glucose uptake, b) This effect of simvastatin is not rescued by addition of mevalonate suggesting it is unrelated to cholesterol biosynthesis, c) Simvastatin does not inhibit pioglitazone mediated glucose uptake pathways but specifically inhibits insulin signaling by IRS-1 phosphorylation, d) The effect may be mediated by buildup of FFA in the cell by simvastatin inhibition of HMG CoA Reductase.

Based on our data we hypothesize that simvastatin may cause insulin resistance through a novel fatty acid based mechanism independent of its cholesterol lowering effects. We hypothesized that by blocking HMG CoA Reductase, simvastatin may lead to accumulation of acetyl CoA, a precursor of fatty acid synthesis. Fatty acids are synthesized from acetyl-CoA and malonyl-CoA precursors. So a potential consequence of treatment of cells with this drug is that it could lead to intracellular buildup of fatty acids.

Our proposed mechanism of statin induced insulin resistance is different from what has been reported by others^21^,^36^. The major difference between our observations and others who have explored the mechanism of statin induced insulin resistance could be due to the fact that in such studies adipocytes and pancreatic beta cells were used where as we used muscle cells as a model^28^. These data implicate inhibition of β-cell glucose transporters, delayed ATP production, pro-inflammatory and oxidative β-cell effects, inhibition of calcium channel-dependent insulin secretion, and β -cell apoptosis^27^. Such mechanisms are not necessarily tied to HMG CoA Reductase inhibition. It is important to point this as the statin’s effect on insulin resistance has been reported in patients with many distinct statins suggesting that the effect is tied to their mechanism of action i.e HMG CoA Reductase inhibition and is not an off target effect.

Another publication has summarized the various putative mechanisms by which statins may cause insulin resistance^36^. The discussion suggested/proposed mechanisms related to direct statin’s effects i.e, decrease in cholesterol biosynthesis related molecules such as coenzyme Q10, farnesyl pyrophosphate, geranylgeranyl pyrophosphate, and dolichol by statin treatment may lead to reduce intracellular insulin signaling. Similarly another possibility is that statins could interference with insulin signaling pathways via inhibition of phosphorylation events and reduction of small GTPase action was also proposed. While all of these are possible mechanism, we propose that FFAs based mechanisms that we demonstrate are equally likely to occur. To this end we do find that in several experiments that manipulation of cellular FFAs content or exogenous FFAs addition appears to be a component of the impact of statins on insulin signaling.

Our proposed mechanism may be only one of many mechanisms by which statins therapy increases the risk of diabetes. Nevertheless it is important to consider mechanism based on muscle cells since defects in glucose uptake by muscle alone can cause insulin resistance and diabetes. The mechanism proposed here will now need to be further corroborated in human studies using muscle biopsies’ of statin treated patients. A robust understanding of the mechanism will help to design strategies to prevent statin induced insulin resistance.

## Materials and Methods

### Cell culture and chemicals

L6 cells (ATCC CLR-1458; Rockville, MD) were maintained in Dulbecco’s modified Eagle’s medium (DMEM) supplemented with 10% Fetal Bovine Serum (FBS) and 100 μg/mL penicillin and streptomycin, at 37 °C in an atmosphere of 5% CO_2_. When L6 myoblasts reached confluence, the medium was switched to the differentiation medium containing DMEM and 2% horse serum, which was changed every alternative day. After 4 additional days, the differentiated L6 cells had fused into myotubes. L6 myotubes were used for treatment as indicated in figures. All the chemicals were bought from Sigma (Sigma, MO, USA) or otherwise stated.

### 2-NBDG glucose uptake

2-(N-[7-nitrobenz-2-oxa-1, 3-diazol-4-yl] amino)-2-deoxyglucose (2-NBDG) (Molecular Probes-Invitrogen, CA, USA) has been used to assess glucose uptake in L6 myoblasts^37^. 50,000 cells/well in 24 well-plates were seeded and differentiated. Post differentiation (myotubes formation), cells were treated with Simvastatin (1 μM) or Atorvastatin (1 μM) or Palmitatic acid (PA, 150 μM) or Pioglitazone (10 μM) or mevalonate (500 μM) or C-75^35^ (40 μg/mL) or PKC pan inhibitor (Bisindolylmaleimide^31^ I, 1 μM) or GÖ6983 (PKC inhibitor 1 μM^38^). Cells were kept in glucose free medium for half an hour before insulin stimulation. Cells were stimulated with 100 nM insulin for 5 and 10 min and incubated with 10 μM of 2-NBDG for 15 min. Reaction was stopped by washing with cold 1X-PBS and the cells were lysed in 0.1% Triton-x 100. The fluorescence intensity of cells containing 2-NBDG was measured using a Wallac 1420 multimode reader with excitation at 485 nm and emission at 535 nm. Values were normalized to the corresponding protein content and expressed relative to control (DMSO) without insulin treatment which was considered as 1.

### Western blotting

After completion of respective treatments, myotubes were scraped and cellular protein lysate was prepared using radio-immunoprecipitation assay (RIPA) lysis buffer. Protein matched lysate were resolved on to 10% SDS–polyacrylamide gel, transferred to PVDF membrane (Millipore, MA, USA), blocked with 5% nonfat milk in TRIS buffered saline (TBS; 10 mM TRIS (pH 8.0), 150 mM NaCl) for 1 hour and probed phospho-IRS-1(Ser^307^) (1:1000, Cell signaling, CA, USA), phospho-IRS-1(Ser^616^(h)/Ser^612^(m)) (1:1000, Cell Signaling, CA ,USA), phospho-IRS-1(Tyr^612^(h)/Tyr^608^(m)) (1:1000, Cell Signaling, CA ,USA), total IRS-1(1:1000, Cell Signaling, CA ,USA), pAkt (Akt Ser 473) (1:2000, Cell signaling, CA, USA), total AKT (1:1000, Cell signaling, CA, USA) and total Actin (1:3000, SantaCruz Biotechnology, CA, USA) using specific antibodies and appropriate HRP-conjugated secondary antibody. The chemiluminescence signals were captured on photographic films using an enhanced chemiluminescence detection system (Amresham, IL, USA).

### Real-Time PCR

For qPCR, reverse transcription and gene amplication for FAS, ACC1 and SREBP2 was performed as reported earlier^39^. The mRNA expression was normalized to corresponding 18S rRNA with the values for control arbitrarily set to 1. All the experiments were performed in triplicates.

### Free Fatty acid (FFA) and Cholesterol quantification

The levels of FFA and Cholesterol were determined using Sigma kit (FFA: MAK044 and Cholesterol: MAK043, Sigma MO, USA). Briefly, cells were lysed in 1% Triton X-100 in chloroform (w/v). The samples were centrifuged at 13000 × g for 10 min to remove insoluble debris. Organic phase was collected and allowed to air dry on 50 ^o^C dry bath for 20 min. Samples were vacuum dried for 30 min to remove traces of chloroform. The dried lipids were resuspended via vortexing in fatty acid assay buffer and were further quantified using manufacturer’s instruction. The values are reported as μM/million cells^40^.

### Statistical Analysis

Values were expressed as mean±SD. For comparison between 2 groups, the unpaired Student’s *t* test was used. For comparisons of 2 or more groups Two-Way ANOVA was used followed by Bonferroni’s post-hoc analysis, *p *< 0.05 was considered as significant.

## Additional Information

**How to cite this article**: Kain, V. *et al.* Simvastatin may induce insulin resistance through a novel fatty acid mediated cholesterol independent mechanism. *Sci. Rep.* **5**, 13823; doi: 10.1038/srep13823 (2015).

## Supplementary Material

Supplementary Information
