# Supplementary material for: Simvastatin may induce insulin resistance through a novel fatty acid mediated cholesterol independent mechanism
Source: Sci Rep. 2015 Sep 8;5:13823. doi: 10.1038/srep13823 (PMC4561915; doi:10.1038/srep13823)

# Simvastatin may induce insulin resistance through a novel fatty acid mediated cholesterol independent mechanism

**Vasundhara Kain*[£1], Bandish Kapadia*[1], Parimal Misra*[¶]and Uday Saxena**[¶]**

*Department of Biology, Dr. Reddy's Institute of Life Sciences, University of Hyderabad Campus, Hyderabad, Andhra Pradesh, India,** Kareus Therapeutics, SA, Switzerland

[¶] Correspondence and request for materials should be addressed to PM (parimalm@drils.org) or US (usaxena@ kareustherapeutics.com).

[£Current address]Division of Cardiovascular disease, School of Medicine, The University of Alabama Birmingham, Alabama, USA.

[1]Both authors contributed equally to this work.

Supplemental Methods

Cytotoxicity assay: L6 Cells were treated with different concentrations of Simvastatin and Atorvastsitn for 48 h and then incubated with 5 mg/ml MTT for 4 h at 37°C. Medium was then removed and 200 µl of DMSO was added to dissolve the crystal. Absorbance was measured at a wavelength of 490 nm using multiplate reader (Perkin-Elmer, Waltham , Massachusetts, USA).

**Supplemental Figure legends**

Supplemental Figure 1: Differentiation of L6 myoblasts to L6 myotubes

Supplemental Figure 2: MTT assay for assessing the cells viability for L6 cells post treatment of the different concentrations of simvastatin and atorvastatin as mentioned.

Supplemental Figure 3: Densitometric quantification of the blots of Figure 4d

Supplemental Figure 4: Full blot images for the cropped western blot films.

Supplemental Figure 1

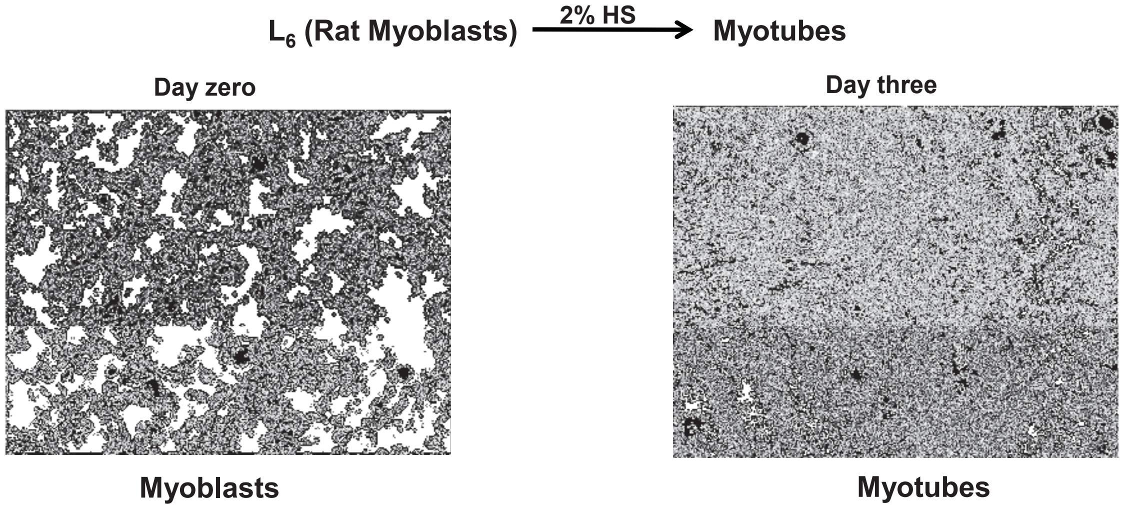

**Supplemental Figure 2**

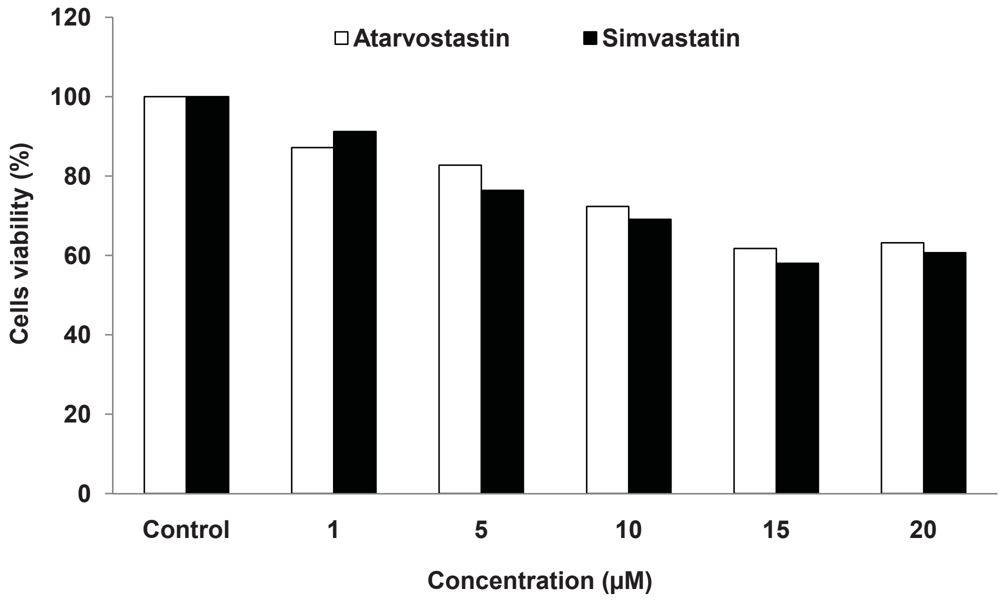

Supplemental Figure 3

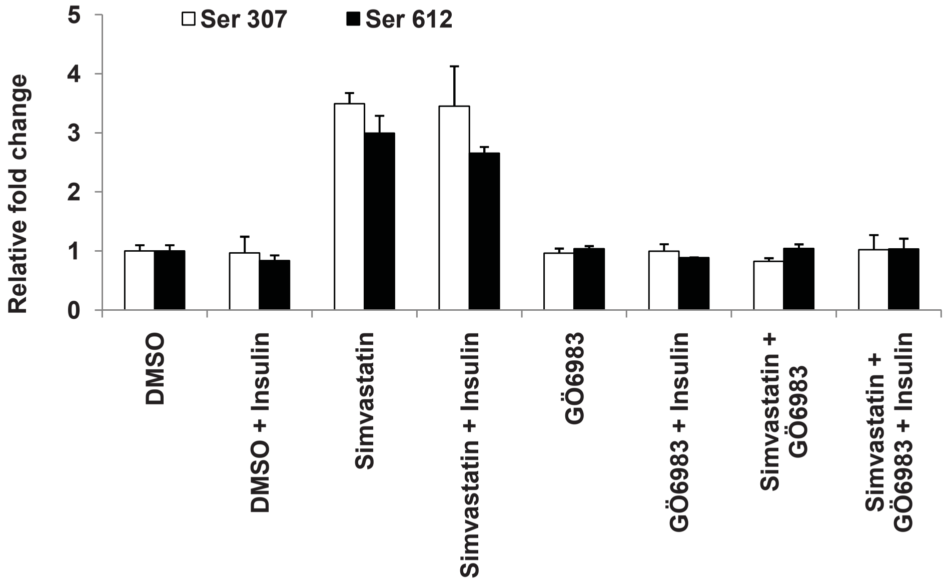

**Supplemental Figure 4**

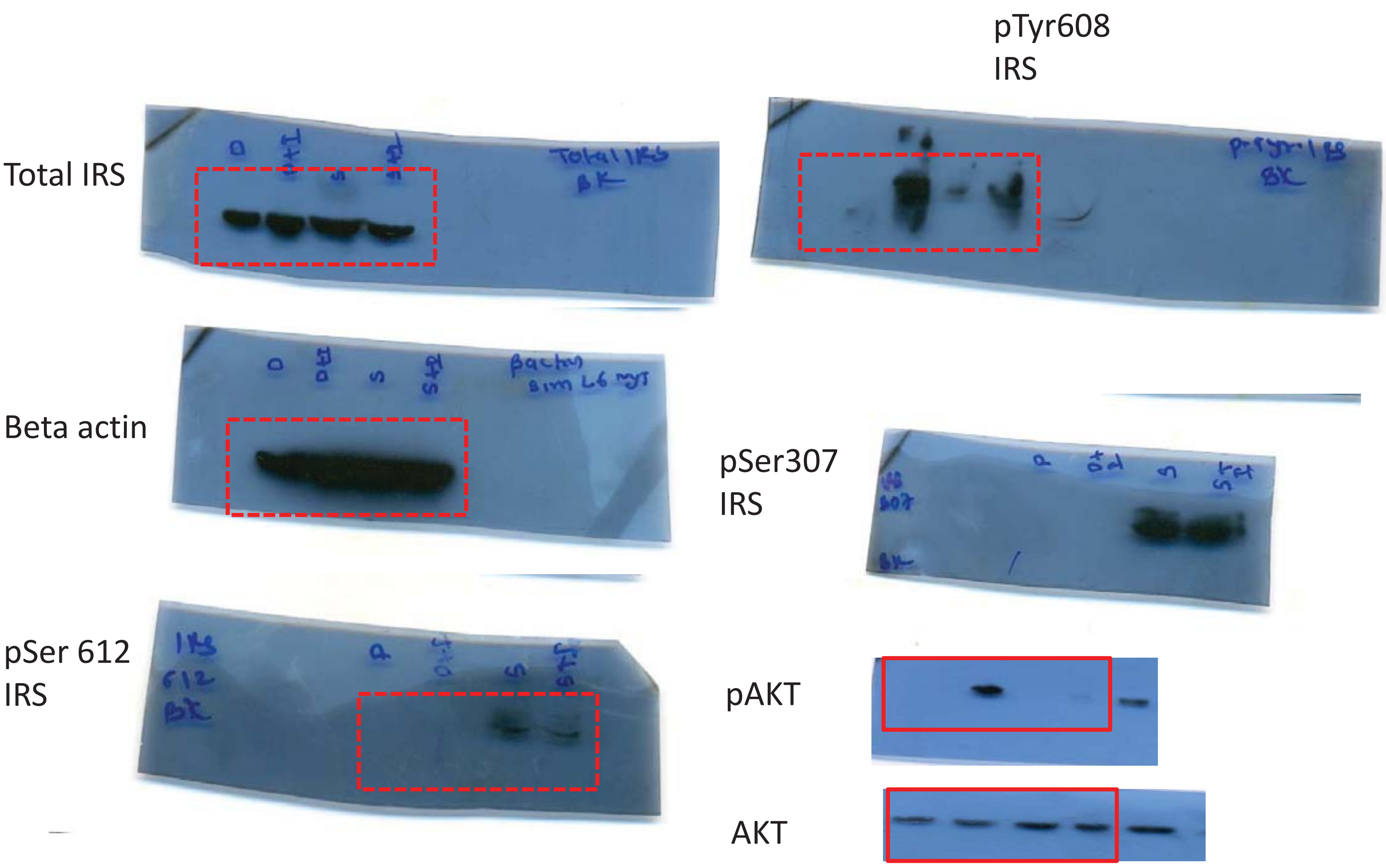

**Supplemental Figure 4**

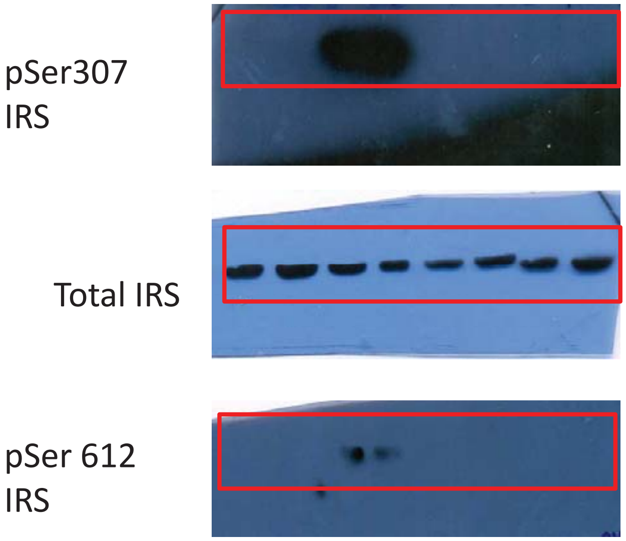

Supplement: Supplementary Information [file srep13823-s1.pdf]
